# 2728. Bronchial Cultures at Time of Transplant Affect Anastomotic Outcomes in Lung Transplant Recipients

**DOI:** 10.1093/ofid/ofad500.2339

**Published:** 2023-11-27

**Authors:** Jesse Veisblatt, Eric Altneu, M Abul Kashem, Yoshiya Toyoda, Peter Axelrod, Aaron D Mishkin

**Affiliations:** Temple University Health System, Philadelphia, Pennsylvania; Temple University Health System, Philadelphia, Pennsylvania; Temple University Health System, Philadelphia, Pennsylvania; Temple University Health System, Philadelphia, Pennsylvania; Temple University School of Medicine, Philadelphia, Pennsylvania; Lewis Katz School of Medicine at Temple University, Philadelphia, Pennsylvania

## Abstract

**Background:**

Anastomotic complications after lung transplant cause significant morbidity and mortality. Estimates of complication prevalence in lung transplant recipients range from 5-20%. Many factors are hypothesized to contribute to airway complications, including the presence of bacteria and fungi at time of transplant. Our aim was to examine the contribution of airway cultures at the time of transplant to airway complications.

**Methods:**

We performed a retrospective analysis of lung transplant recipients who developed bronchial complications at a single, tertiary care center between January 1, 2016 and March 31, 2020. We identified 68 cases with anastomotic complications during this time period, then compared this cohort to 131 cases without bronchial complications. Primary outcomes were airway complications at 30 and 90 days. Please refer to the attached tables for variables of interest.

**Results:**

Of 199 total study participants, 68 (34%) were found to have airway complications. Overall, the most common bacterial isolate was Staphylococcus aureus (72 MSSA and 17 MRSA). The most common fungal isolate was Candida albicans (87). When examining the patients with airway complications, MRSA isolates at time of transplant showed a statistically significant increase in airway complications (p=0.03, OR=3). An increase in airway complications was not seen for patients with other positive bacterial or fungal cultures at 30 days or 90 days.

Microbiological Risk Factors for Airway Complications

**Figure:**
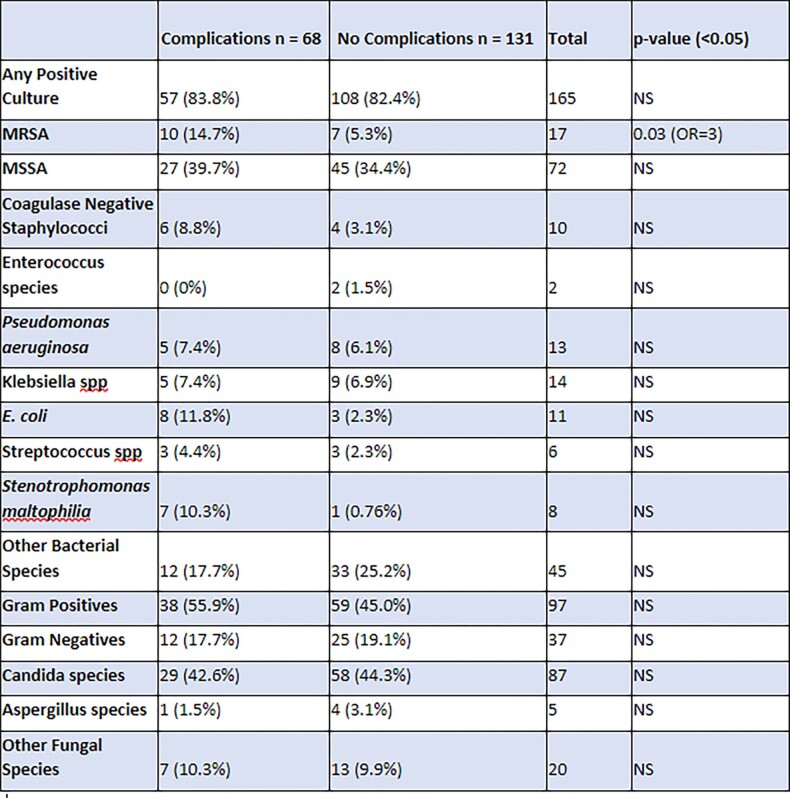

Patient Demographics and Characteristics

**Figure:**
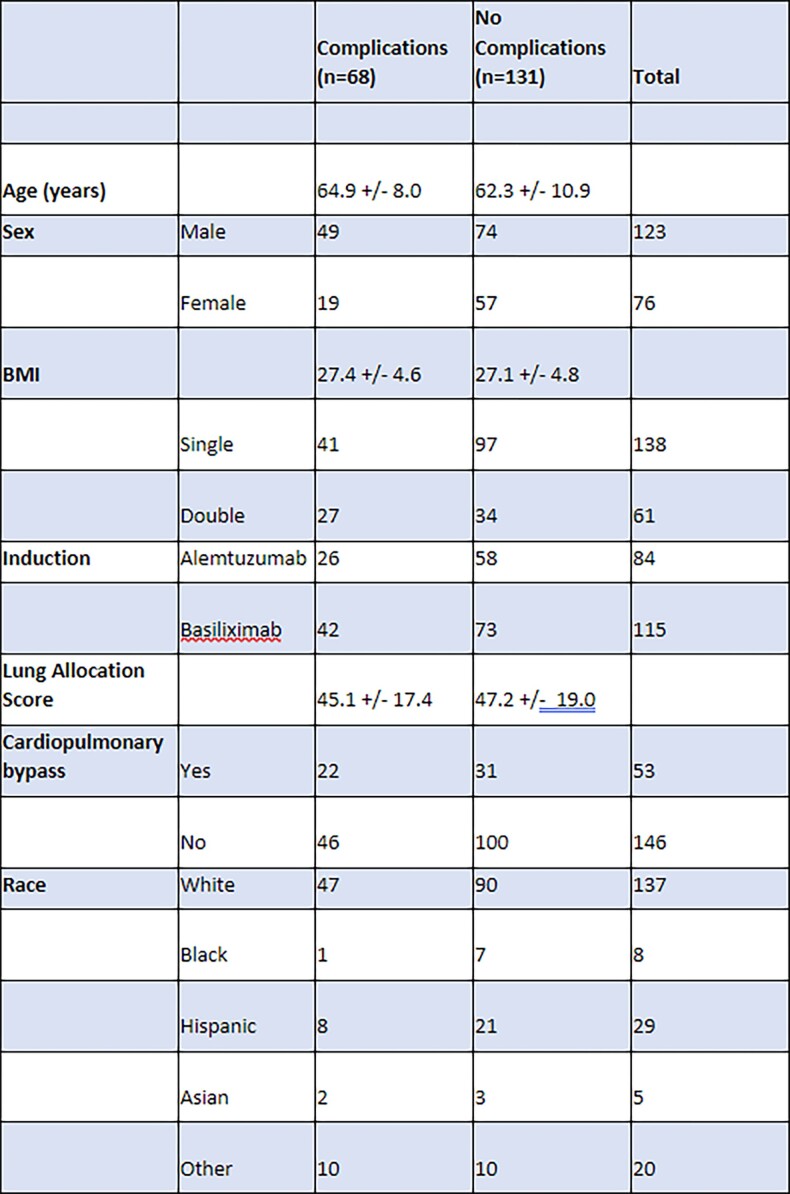

**Conclusion:**

MRSA isolates at time of transplant correlated to increased 30 day and 90 day bronchial complications. This study helps confirm the importance of monitoring airway cultures at the time of transplant. A multi-center study with a larger sample size may be needed to further delineate contributions of specific microorganisms, surgical techniques, as well as perioperative and prophylactic antimicrobials on bronchial complications.

**Disclosures:**

**Aaron D. Mishkin, MD**, Pfizer: Grant/Research Support|Takeda: Advisor/Consultant

